# Monarch-1 Activation in Murine Macrophage Cell Line (J774 A.1) Infected with Iranian Strain of *Leishmania major*

**Published:** 2013

**Authors:** A Fata, MR Mahmoudian, A Varasteh, M Sankian

**Affiliations:** 1 Research Center for Skin Diseases and Cutaneous Leishmaniasis, School of Medicine, Mashhad University of Medical Sciences, Mashhad, Iarn; 2 Department of Parasitology & Mycology, School of Medicine, Mashhad University of Medical Sciences, Mashhad, Iarn; 3 Allergy Research Center, School of Medicine, Mashhad University of Medical Sciences, Mashhad, Iarn; 4 Immunology Research Center, School of Medicine, Mashhad University of Medical Sciences, Mashhad, Iarn

**Keywords:** Monarch-1, *Leishmania major*, J774 A.1, NALP12, Murine macrophage cell line

## Abstract

**Background:**

*Leishmania major* is an intracellular parasite transmitted through the bite of the female *phlebotomine* sand flies. *Leishmania major* is able to escape the host immune defense and survive within macrophages. Modulation of the NF-κB (Nuclear Factor-Kappa B) activation and suppression of the pro-inflammatory cytokines by *L. major* are the main evasion mechanisms that remain to be explored. This study aims to examine the expression level of the Monarch-1 in *L. major-*infected macrophages, as a negative regulator of the NF-κB activation.

**Methods:**

Murine macrophage cell line (J774 A.1) was infected by metacyclic form of *Leishmania* promastigotes at macrophage/*parasite ratio of* 1:10. After harvesting infected cells at different times, total RNA was extracted and converted to cDNA. Semi-quantitative RT-PCR was performed for Monarch-1 by specific primers. Hypoxanthine Phospho-Ribosyl Transferase (HPRT) was used as an internal control to adjust the amount of mRNA in each sample.

**Results:**

Semiquantitive analysis of Monarch-1 mRNA expression level showed a significant expression increase within 6 to 30 hours after *L. major* infection of macrophages when compared to the control macrophages.

**Conclusion:**

Monarch-1 expression level reveals a significant increase in the early phase of macrophage infection with *L. major*, which in turn may suppress IL-12 production in *Leishmania* infected macrophages and deeply influence the relationship between host and parasite.

## Introduction

*Leishmania* is an intracellular parasite lives inside the histiocytes of mammals. It exploits a numer of mechanisms to escape phagocytosis and survive within the macrophages. *Leishmania* suppresses different activities of macrophages including phagocytosis, nitric oxide synthesis, IL-12 production and MHC class II presentation (1, 2). Several parasites including *L. major* and *Toxoplasma gondii*, affect key components of *NF-κB* signaling pathway which participates in innate and acquired immune responses (3, 4). Since, the majority of these mechanisms is not well known, immunization and preparation of vaccine against leishmaniasis has not been successful.

Monarch-1 activation seems one of the possible mechanisms that could account for NF-κB inhibition and suppression of IL-12 production. The Monarch-1 molecule, also known as PYPAF7, NALP12,PAN6, is one of the Nucleotide-binding and leucine-rich repeat-containing receptors (NLRs) family which contains an N-terminal effector PYRIN domain and has anti-inflammatory or immunosuppressive functions (5). Monarch-1 is often expressed on the myeloid cells including monocytes and granulocytes and regulates non-canonical and **canonical** NF-κB activation (6). This regulatory effect is likely achieved by degradation of NF-κB inducing kinase (NIK) via proteasome-dependent and independent pathways. In this context, some researches imply that Monarch-1 suppresses production of pro-inflammatory cytokines and chemokines (7). It has been also reported that this molecule may interact with HSP90 and “adaptor-like” proteins, such as Fas-associated factor 1 (FAF-1) which links innate immunity and apoptosis signaling (8). Recently, it has been indicated that Monarch-1 also regulates migration of dendritic and myeloid cell into the cutaneous inflammation sites (9).

To examine the possible role of the Monarch-1 in *Leishmania* survival inside the macrophages, this study examines Monarch-1 (NALP12) mRNA expression in *Leishmania*-infected mouse macrophage cell line.

## Material and Methods

*Leishmania major, strain* MRHO/IR/75/ER, obtained from Pasteur institute, Tehran. The parasites transferred into biphasic NNN medium containing 250 IU/ml penicillin and 250 µg/ml of streptomycin. After 3 days, a fresh smear from liquid phase of biphasic culture was obtained and examined under low power light microscope. The promastigotes were counted using the hemocytometer cell counting chamber. After parasite count reached to 2x10^6^ /ml, the promastigotes were transferred to the culture tubes containing RPMI 1640 plus 10% Fetal calf Serum (FCS) and kept at 25 °C until their population increased to 7x10^7^ promastigotes/ml. After cultivating of the *L. major* promastigotes for 3 to 4 days in liquid medium, *L. major* promastigotes transform from a less infective procyclic form to a highly infectious metacyclic promastigote during the stationary phase of growth. The mouse macrophage cell line, J774 A.1, was obtained from the cell bank of *Pasteur institute* of Iran. The macrophages were cultured into a flask containing RPMI 1640, 15% FCS, streptomycin 100 mg/ml and penicillin 100 IU/ml, and kept at 37 °C in an incubator containing 5% CO_2_. After growth of macrophages, they were trypsinized and seeded into six-well plates.

To infect macrophages by metacyclic form of promastigotes, 1 × 10^6^ cells/well macrophages plated in six-well plates and kept at room temperature for 24 hrs. All wells with macrophage monolayers were infected with promastigotes in stationary phase using 10 parasites per cell and incubated at 37 °C for 2 h. To eliminate free swimming promastigotes from the cultures, the supernatant was discarded and the macrophages were washed 3 times gently by RPMI 1640. Then, 5 ml fresh RPMI 1640 was added to each culture chamber and incubated for 6, 18 and 30 hours (10–12). Each incubation time carried out in quadruplicate wells *simultaneousl*y. To the indicate macrophage infection, Intracellular parasites were enumerated by invert phase contrast microscopy. Non infected-macrophages also incubated over the same time in quadruplicate wells. After the incubation periods, the macrophages were trypsinized and harvested from the plates. RNA extraction was performed by TriPure isolation reagent (Boehringer Mannheim) according to the manufacturer's instruction. The extracted RNA was reverse transcribed to cDNA by cDNA synthesis kit (Fermentas Life Science, Lithuania). Then, semi-quantitative RT-PCR was performed for Monarch-1 by specific primers. PCR primer sequences were based on the Ensembl database (http://www.ensembl.org) and were designed by Gene Runner (Hastings Software Inc., Hastings, NY) and Primer Premier 5 (Premier Biosoft Inc). BLAST analysis against mouse mRNA was performed, using Ensembl database, to test the specificity of the primers (Table 1). To normalize the interest gene, Hypoxanthine Phospho-Ribosyl Transferase (HPRT) was used as an internal control to adjust the amount of mRNA in each sample.

**Table 1:** Primers used for RT-PCR of Monarch 1 and HPRT

Genes	Primers	Product size	T_op_	Accession No.
Monarch-1 (NALP12)	F: 5’ – GTACCAACTCCAACCTGATCG– 3’ R: 5’ –GAAGTAGAGGCCAGATTTGC– 3’	518 bp	59 °C	CCDS51963.1
HPRT	F:5’ – CGTCGTGATTAGCGATGATGAAC– 3’ R:5’ –TCACTAATGACACAAACGTGATTC– 3’	609 bp	59 °C	CCDS40972.1

The intensity of obtained bands was determined by Kodak 1D software (Eastman Kodak Company, Rochester, NY). Graph Pad Prism-5 (Graph Pad Prism Software Inc) was used for the statistical analysis, Kruskal-Wallis test, and graph drawing.

## Results

The result of PCR optimization experiments for HPRT gene showed that the best annealing temperature was 59 °C for 34 cycles to obtain a sharp band in the exponential phase of amplification. In this study, the expression level of Monarch-1 mRNA was evaluated by semi-quantitative RT-PCR in *L. major* treated and untreated J774 A.1 cell lines.

**Fig. 1:**
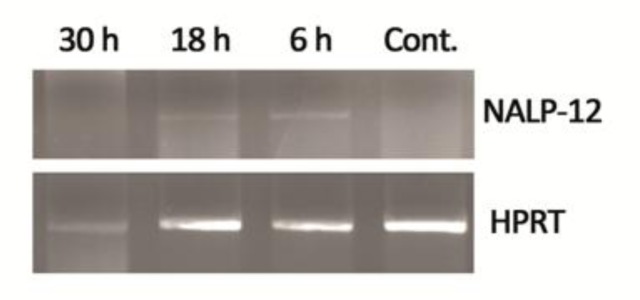
Semi-quantitative RT-PCR analysis of Monarch-1 expression in *Leishmania major*-infected and non-infected cells after 6, 18 and 30 hours

**Fig. 2:**
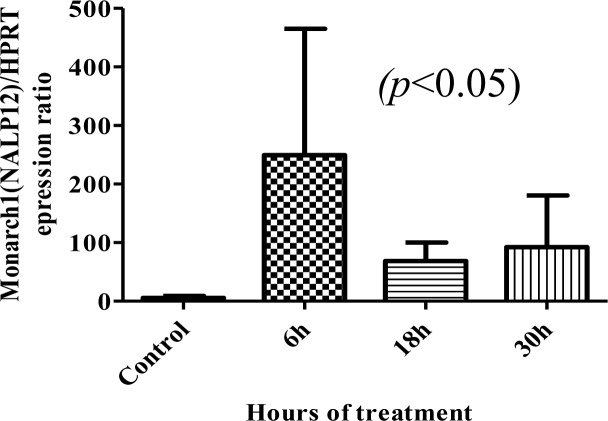
Comparison between the average of four separate experiments measuring mRNA Monarch-1 expression in *Leishmania major*-infected (6, 18 and 30 hours) and non-infected cells. Bar graph indicates the mean ± S.E.M

Considering the relative expression of Monarch-1 normalized to the HPRT expression level, Monarch-1 mRNA level showed a significant increase within 6 to 30 hrs after infection with promastigotes, while no expression were detected in the control samples (Figs. 1 and 2).

## Discussion

In the mutual relationship between parasite and macrophage, there are various tricks for parasite survival in the macrophages. *Leishmania* evokes different strategies to subvert macrophage functions. One of them is modulation of the Rel/NF-κB transcription factor activity, which plays a pivotal role in several processes of immune and inflammatory responses (13, 14). In this study, findings indicate a significant expression of Monarch-1 in *Leishmania* infected macrophages. It seems one of the possible mechanisms that could account for NF-κB inhibition and suppression of IL-12 production.

Previous studies showed that *Leishmania amastigotes* prevent translocation of p50/p56 into the nucleus with an unknown mechanism (15). It has been also demonstrated that in the peritoneal macrophage of *Leishmania*-infected mouse, a few NF-κB proteins are bound on the NF-κB binding sites (16).

Previous studies indicated that macrophage suppression, which is induced by *L. major*, are initiated along with attachment of parasite on the macrophage surface. *Leishmania* entrance into the macrophages is mediated through molecules that induce translocation of new described NF-κB complex, p50/C-Re1, into the nucleus, instead of p50/p65. These receptors are not completely defined, but there are some evidences that imply on the role of TLRs in this mechanism (17–20).

In addition to the surface receptors, new family of intracellular receptors, so called NLR, has been found which could be a candidate for interaction with *Leishmania* and modulation of NF-κB activity. NALP12 or Monarch 1 is a regulatory protein of NLR family that has a proven suppressive effect on the NF-κB activity. This molecule exploits several mechanisms to modulate NF-κB signaling pathway, including hyperphosphorylation of the IRAK, degradation of NIK and interaction with other regulatory proteins (5, 21). To our knowledge, the present study is the first report on possible implications of this molecule in the relationship of *L. major* and host.

## Conclusion

Although further analysis will be required to clarify the role of the Monarch-1 in immune evasion by *L. major*, our findings suggest that expression of this molecule in the early phase of macrophage infection with *L. major*, could be an explanation for suppression of IL-12 production in *Leishmania* infected macrophages. In turn, it might deeply influence on the future of the host and parasite relationship.
